# Epidemiology, Public Health, and Public Policy

**Published:** 2009-09-15

**Authors:** James S. Marks

**Affiliations:** The Robert Wood Johnson Foundation

I left the Centers for Disease Control and Prevention (CDC) more than 4 years ago to go to the Robert Wood Johnson Foundation (RWJF). RWJF had gone through a time of substantial rethinking about its role in society and come to the conclusion that the focus should not be grantmaking; it should be creating social change for health. This could just as easily be said about public health — creating societal change to improve the health of the public.

At its core, public policy is the way a society frames what it wishes to become. Does it want all children immunized? Does it want to limit the use of tobacco? Policy doesn't have to be federal legislation or regulation; it can be corporate or local or state. But an organization, a field, or groups that are about social change will find they are often about influencing public policy.

How does this fit with epidemiology and its history, and the history of CDC?

It is unlikely that John Snow would be revered in public health if he had merely studied cholera. His defining moment was when he removed the pump handle from the contaminated well that was the source of the epidemic. That intervention is honored with an award called the Pump Handle Award that is given by the Council of State and Territorial Epidemiologists. Our heroes have been tied to action.

In 1983, giving the Wade Hampton Frost Lecture at the American Public Health Association meeting, Bill Foege, former CDC director, coined the term *consequential epidemiology*. Foege was taking sides in an active debate of the time by saying that epidemiology "is a tool to change the world, not merely to study the world" (unpublished material, 1983). The debate that Foege was speaking to was whether epidemiologists should engage in the political process. Should they advocate solutions about what should happen based on the science or merely do the science and let the advocates and policy makers use the science? Some people said that scientists would lose their objectivity if they took on this more activist role. This discussion has come back, quite strongly, in recent times in the context of the politicization of science. Politics and public policy can be tough business, especially recently.

Bill Foege was of a very different opinion. It was his conviction that public health was *inherently* political, *inescapably* political. Foege argued that public health work occurs in a political context and that, divorced from that context, our science is stillborn, a missed opportunity. To pretend otherwise is self-delusion and a failure of vision and responsibility.

The purpose of this essay is to reaffirm that connection to activism. I state this not despite recent concerns about politicization of science but because of them. We must embrace both 1) activism and commitment to social change as central to public health, and 2) the central purpose of epidemiologic science, which is to find, assess, and confirm truth, regardless of past findings or beliefs. These are different roles, and the space between them is where the real excitement is. Actions are being taken based on the science as it is known today, but the actions will change as the science advances.

We are entering a period of economic pressure that is likely to change the nature of America's medical care, the largest industry in our nation. We now spend about twice as much as other developed countries per capita per year. Despite the importance of biological science as the basis for improvements in diagnosis and therapy, it is impossible to think of major changes in the medical care system that will not play out in public policy changes. The public's health and the societal factors that affect health span a much greater proportion of our economy and our society than even medical care does. This means that public health will have to address issues that have political implications. Yet the science behind what can be done does not mean it will be done.

Our policy makers and the public have not been nearly as committed to the understanding that how our society is organized, what our policies foster or inhibit, what our communities encourage and our institutions support are fundamental causes of good or ill health, just like biological causes, and they warrant study and action. If societal forces are not in alignment, scientific advances stall, and the value realized is a meager fraction of its potential. Scientific understanding about any disease that is not widely applied to people in need is ultimately wasted. Similarly, widespread application of practices and policies that have no scientific evidence of effectiveness are just as futile. Scientific discovery and widespread application must never be separated.

This is a defining tension for all of public health. Scientists never feel they know enough to act. Practitioners and activists say the health problems are so significant we must act now; we can't wait for the science to be finalized. Both are right. Organizations only responsible for research will worry less about whether the findings are widely applied or are feasible and practical. Organizations only responsible for programs will likely hold to outmoded ways that are ineffective if their staff and clients like the program. Managed well, the combined responsibilities make both science and program better.

## Role of Epidemiologic Science

Most people think only about the biologic mechanisms as the fundamental causes of good or ill health, hence of medical care as the central intervention for health. What many people fail to realize is that the likelihood of initially developing a disease or being injured has little to do with access to the medical care system. This is especially true regarding population differences. Initially developing an illness or suffering an injury is more related to such things as whether people smoke, what and how much they eat, how active they are, what toxins or microbes they are exposed to, and whether their neighborhood or worksite is safe.

The *Tao* *Te Ching*, an ancient Chinese text about how society operates, says that the leader should guide quietly and unobtrusively so that the decisions that are made are felt by the people to be ones they came to. If a leader aggressively pushes decisions, those will be met with strong opposition. Epidemiologists' place in society can be very much like that of the Taoist leader: a position of tremendous power and influence, but only if it is guided carefully and not forced.

More than any other group in health, epidemiologists decide how to measure the health of the nation. They choose what questions are asked and analyzed, what differences are important, serious, and worthy of comment, even alarm. If you do not ask the questions, the only answer is silence.

It is a position of huge responsibility and equally large opportunity to profoundly affect the national debate about health. At its most powerful, epidemiology is about asking questions of the most fundamental but often hard to measure, aspects of health, wellness, and even happiness and life satisfaction that comprise the fullness of the World Health Organization definition of health. When framed in the context of disparity, it opens up how that vision of health ties to our nation's highest ideals of equality of opportunity.

Also powerful is how the epidemiologist frames findings. Articles published during the early stages of the obesity epidemic got little attention, even though the upward trend was clear. In the late 1990s Ali Mokdad, a CDC scientist who was then running the Behavioral Risk Factor Surveillance System (BRFSS), had the idea to use maps, which have become well known in the field, to illustrate the change in obesity in the nation.

A series of color-coded maps showed changes in obesity rates over the years in each US state. The rapidity of the increases, coupled with geographic framing, was a visually powerful representation of the increase in prevalence. Those images, probably more than any other, caused the media and the public to take notice. That art to frame and energize debate often goes unrecognized, but it is another lever that epidemiologists have — the power of display and presentation and framing as leading without forcing.

Lastly, epidemiology plays a role in evaluating the effect of policies implemented. This study of policy is often a very tricky issue. Frequently, conducting a randomized trial is nearly impossible in public policy, where nonrandom natural experiments are sometimes the best possible evidence. Societal decisions often have to be made on the basis of evidence that is not as controllable as a randomized trial of medication effectiveness. The role of epidemiology is to be that honest broker of the science, regularly improving understanding and identifying problems and risks.

Public health practice and public policy are about applying what is known and possible. It is much like an oncologist may treat a cancer patient, knowing that the treatment has great limitations and that new science will come, but he must act now to help the patient using what is available. The public health practitioner must act on behalf of the people and must be prepared to change course as the science improves. This means that epidemiologists must be free to speak about their findings, and they must be true to what the data show. Public health and the rest of the public process, including policy, will use those findings, more or less within a societal and political context.

To return to the cholera story, it turns out that John Snow did not actually remove the pump handle from the well. Steven Johnson, in his excellent history of the cholera epidemic, *The Ghost Map* (1), writes that on September 7, 1854, John Snow presented his findings to the board of governors of St. James Parish. After much discussion, the board voted to close the Broad Street well, despite its reputation for clean water, because the evidence was so strong. Snow's place of honor in our field is thus even more warranted as an early interplay between epidemiologic science, public policy, and health. At its best, epidemiology is persuasive.

## Why Public Health and Prevention?

During the past decade we have added approximately $1 trillion annually to our medical care budget and lost ground in life expectancy and infant mortality relative to other countries of similar economic development. Simply put, they are getting healthier, faster than we are, despite our great increase in funds. Health care reform is essential, but as a nation we will have to embrace other ways to improve health and to rein in the rate of increase in the growth in medical care costs. Public health and prevention should be part of that solution.

Public health practitioners tend to think of health as the outcome that people want, so we talk about the importance of getting a flu shot or not smoking to prevent illness or death. But what do people really seek when they aspire to a healthier life? I believe that what people really want is a meaningful, satisfying life of doing things they value and enjoy.

Good health is not the end but the means to an end. Health is a crucial foundation on which people have their best chance to build an enjoyable and satisfying life for themselves and their families. The political will of our leaders is often built the same way, as are the priorities of business and industry. As political leaders think about major program or policy needs, they think about what is most important for their state or city. Usually that is about helping it become economically stronger, a better place for families to live, work, and play. For those leaders, recognizing that health is a means to these ends and a measure of how good a place is to raise a family helps connect it to the things they find valuable.

## Social Justice

We need to work harder to achieve social justice. The philosophy of public health is social justice. Our responsibility is to do what we can to reduce or eliminate disparity.

In 1986 Bill Foege spoke against the backdrop of circumstances that were not all that different from our own: an economy just coming out of recession, ballooning deficits, and malfunctioning markets. He said:

. . . because of the way the market system works, our special clientele . . . will continue to be the poor, the homeless, the unimmunized, the hungry, the addicted, and those who simply find the system overwhelming. . . . Let me assure you, we will survive any crisis that involves funding, political support, popularity, or cyclic trends, but we can't survive the internal crisis, if we become provincial, focus totally on the short term, or if we lose our philosophy of social justice (2).

My final challenge, a challenge that only our nation's practicing public health epidemiologists can accept, is to report injustice and disparity regardless of your field of study. Public health's overarching goal is to reduce or eliminate differences in health and, ultimately, what gets measured *is* what gets done. That means you must measure — justice.

## References

[1] Johnson S (2006). The ghost map.

[2] Foege WH (1987). Public health: moving from debt to legacy. Am J Public Health.

